# Ecchymosis on the penis, scrotum and pubis

**DOI:** 10.11604/pamj.2017.28.252.14366

**Published:** 2017-11-22

**Authors:** Glauber Voltan, Fred Bernardes Filho

**Affiliations:** 1 Independent Radiologist Consultant, Juína, Mato Grosso, Brazil; 2 Dermatology Division, Department of Medical Clinics, Ribeirão Preto Medical School, University of São Paulo, Ribeirão Preto, Brazil

**Keywords:** Penile diseases, male urogenital diseases, scrotum

## Image in medicine

A 52-year-old male patient with a mechanical prosthetic valve, in use of the combination of aspirin and warfarin, presented to the emergency department after 12 hours of a blunt injury of erect penis during sexual intercourse. On physical examination there was swelling and subcutaneous hematoma extending over the proximal penile shaft, the scrotum and the pubis with a dorsal-left sided deviation of the penis. The scrotum was swollen and painful on physical examination and the testicles were palpable with difficulty due to the pain. The patient denied any voiding difficulties or haematuria and had neither blood at the meatus nor palpable bladder. On the basis of the history and physical examination, a diagnosis of penile fracture and urethral trauma was made. The patient was brought immediately to the operating room for retrograde urethrography and surgical exploration. Although highly underreported, penile fracture is uncommon urologic injury. These injuries attract a lot of psychological, physical, functional, and emotional distress on the patient. The diagnosis of penile fracture is largely clinical with a definite history of trauma, a “crack” sound and rapid detumescence of the erection. Depending on whether the Buck's fascia has been breached, the hematoma will track to the base of the shaft or spread to the perineum, scrotum (butterfly sign) and lower abdominal wall. If the fracture involves the urethra, blood will extravasate through the urethra. Urethral bleeding or hematuria or voiding symptoms suggest associated trauma to the urethra.

**Figure 1:**
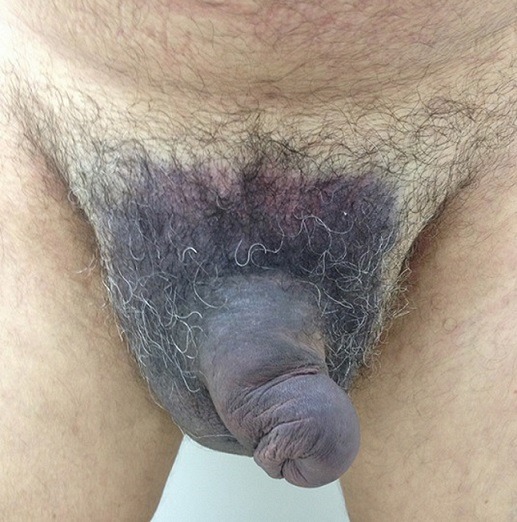
Penile swelling and ecchymosis

